# TICD: a novel thermal imaging cats' dataset for non-invasive health monitoring

**DOI:** 10.3389/fdgth.2025.1650223

**Published:** 2025-08-15

**Authors:** Mohammad Abdulghafar, Adnan Nadeem, Anas Alhindi, Hudhaifa Gburi, Saifullah Abdulghaffar, Emad Nabil

**Affiliations:** Faculty of Computer and Information Systems, Islamic University of Madinah, Madinah, Saudi Arabia

**Keywords:** thermal imaging, cat health monitoring, non-invasive health monitoring, classification, machine learning, deep learning

## 1 Introduction

Digital health technologies have revolutionized the healthcare landscape by providing non-invasive, efficient, and scalable solutions for disease monitoring and early detection. Through the integration of sensors, imaging tools, and artificial intelligence (AI), digital health tools facilitated continuous and remote assessment of physiological and pathological conditions, resulting in better outcomes, lower costs, and improved patient compliance (1).

Among these tools, thermal imaging stands out for its ability to capture heat distribution across the body, offering insights into underlying physiological changes such as inflammation, infection, or circulatory irregularities. Unlike conventional diagnostic methods, thermal imaging is contactless, safe, and real-time, making it particularly useful in scenarios where minimizing stress is critical (2, 3).

In animal digital healthcare, thermal imaging has emerged as a promising tool for monitoring and diagnosing a range of conditions. Research has shown its utility in identifying musculoskeletal injuries in horses (4), mastitis in dairy cows (5, 6), and inflammation or fever in dogs (6). The authors in (7) reviewed the applications of thermal imaging in biomedical research for animals. The study presents that infrared thermography (IRT) can evaluate tissue viability, identify inflammation areas, and detect burn injuries in animals. In another review (8) investigated the use of thermal image analysis for disease detection in livestock. They suggest that IRT can detect diseases that affect the blood supply or cause inflammation, such as digital dermatitis, laminitis, and foot-and-mouth disease (FMD). They also highlight the inconsistencies in practices and the use of variable parameters in existing studies in the literature related to the use of thermography for animal disease detection. The non-invasive nature of thermal images is particularly beneficial for pets, as it minimizes stress and improves compliance during monitoring procedures.

Cats, in particular, present unique diagnostic challenges due to their subtle signs of illness and tendency to mask symptoms. The use of thermal imaging in cats' health monitoring holds strong potential, as it enables pet owners to detect localized heat changes indicative of injury, infection, or systemic illness without the need for visiting veterinary doctors.

### 1.1 Gap analysis

While thermal imaging has gained attention in veterinary diagnostics, there is a noticeable lack of publicly available datasets focusing on feline health assessment. Existing datasets and research primarily focus on livestock or dogs, leaving a gap in tools and data tailored to cats. To our knowledge, there is currently no open-access dataset specifically comprising thermal images of cats annotated for health monitoring purposes. This scarcity limits the development and validation of AI models aimed at the automatic detection or classification of feline health conditions using thermal data.

### 1.2 Contribution

To address this gap, we introduce TICD (Thermal Imaging Cats' Dataset), a novel dataset designed for non-invasive health monitoring of cats using thermal and digital imaging. TICD consists of 1,899 thermal and 527 digital images collected from 94 cats across various locations in Saudi Arabia, covering both indoor (controlled) and outdoor (uncontrolled) environments.

The dataset includes 81 images of healthy cats and 13 of symptomatic cats, providing a foundation for binary classification (healthy vs. sick) and potential future modeling of specific conditions. The digital images complement the thermal ones by documenting visible anomalies such as posture, wounds, or swelling.

The difference in the number of thermal images (1,899) vs. digital images (527) in the TICD dataset reflects their distinct purposes. Thermal images are the primary data source for analysis and model training. It is essential to collect more than one thermal image, as signs of heat may appear partially from one angle and more clearly from another. In contrast, digital images are included for documentation. Each digital image visually confirms the physical condition of the corresponding cat and provides a reference that links the thermal signature to the cat's visible appearance.

TICD provides researchers with a valuable resource for developing AI-driven diagnostic tools, benchmarking classification models, and conducting comparative analyses across various environmental conditions. By making TICD available, we aim to foster advancements in feline digital health and encourage further investigation into non-invasive diagnostic technologies.

### 1.3 Report organization

The remainder of this report is organized as follows: Section 2 presents the Materials and Methods used in creating the TICD dataset, including the methodology, data collection protocol, instrumentation, and dataset annotation. Section 3 demonstrates the potential of TICD for developing health classification models for cats. Section 4 provides a TICD baseline implementation and results for initial validation. Section 5 presents a discussion of the dataset's key aspects.

## 2 Materials and methods

### 2.1 Methodology

**Figure 1:**
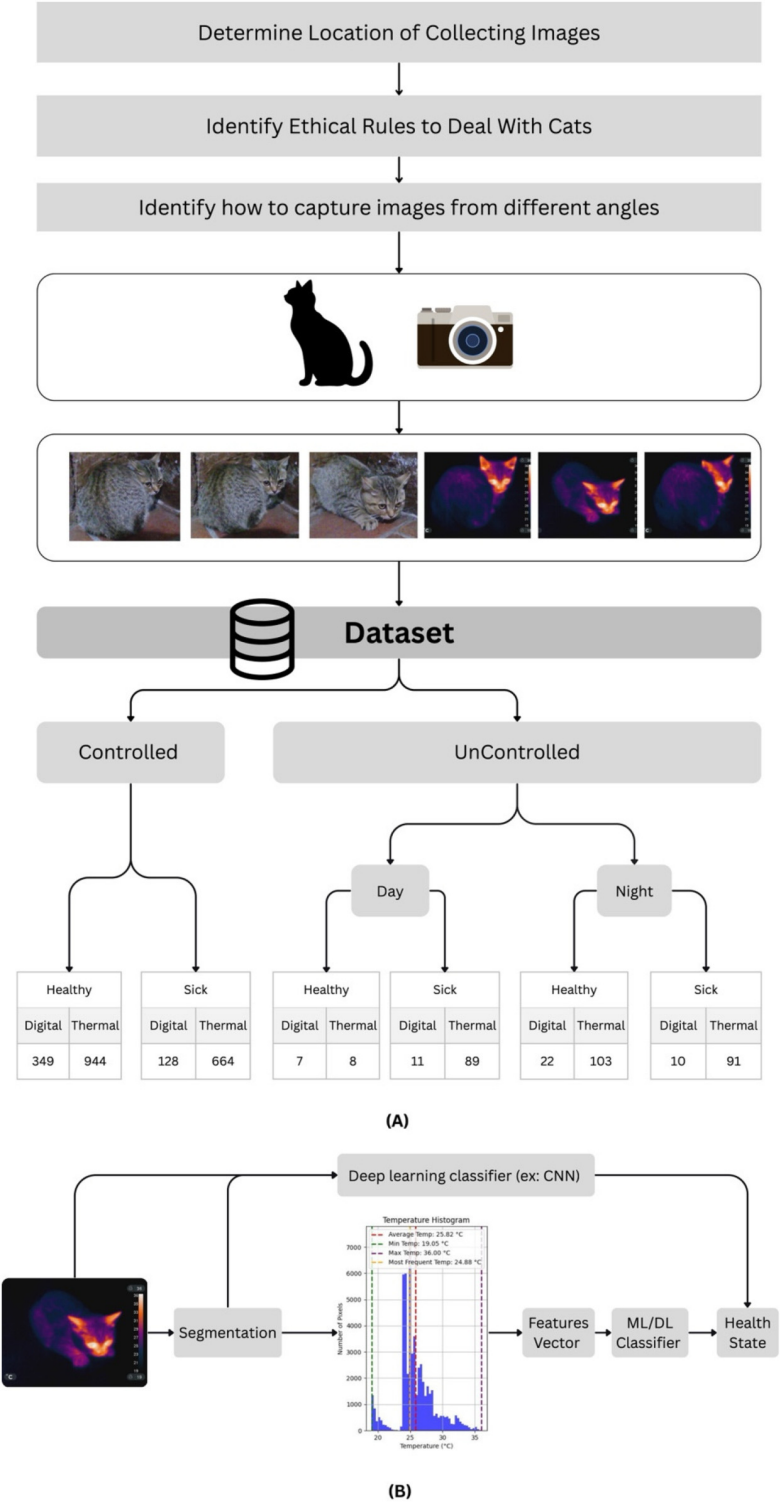
The dataset organization and its utilization **(A)** represents the structure of the dataset. **(B)** Shows an example of the utilization of the dataset in classification.

The process of constructing the TICD dataset followed a structured pipeline as shown in Figure 1A. The methodology involved selecting diverse collection locations, following ethical imaging procedures, and capturing images from multiple angles using thermal and digital cameras. These images were gathered across both controlled and uncontrolled settings to ensure environmental diversity and robustness in dataset composition.

### 2.2 Data collection protocol

We collected data over a six-month period, from September 2024 to March 2025, across two environmental settings:

• Controlled indoor environments such as veterinary clinics (*Nabd Al Farah*, *Al-Asr Veterinary Doctors Center*) and pet-related facilities (*Black Forest Pets and Supplies, Cat Lounge*).
• Uncontrolled outdoor environments in public areas of Al Ruwais (Jeddah) and Al-Usbah District (Medina), which varied in lighting, ambient temperatures, and background thermal noise.

Each cat image was taken from multiple angles using both traditional digital cameras and thermal imaging.

### 2.3 Instrumentation and setup

The thermal images in the TICD dataset were captured using a Seek Shot thermal camera with a resolution of 640 × 480 pixels, capable of capturing both thermal and digital images. The camera has a temperature sensitivity range of −40°C to 330°C, making it suitable for non-invasive surface temperature measurements. Thermal images are saved in JPEG format, embedded with thermal color maps representing temperature gradients in Celsius—red/yellow hues indicating warmer regions and blue hues indicating cooler areas.

To ensure the safety and comfort of the animals, we captured images from 90 cm to one meter. Given the natural behavior of cats—who tend to move frequently and unpredictably—images were taken from multiple angles. This variability was intentional, aiming to simulate real-world, non-laboratory conditions and enhance the robustness of machine learning models trained on the dataset. While we maintained as much consistency as possible in the imaging setup, some minor variations in lighting and angles were present. These variations reflect the dataset's goal of supporting practical and flexible thermal imaging applications. In parallel with the thermal imaging, high-resolution RGB digital images were captured to document visible physical conditions such as abnormal posture, wounds, or fur loss. These digital images serve as complementary data, providing visual cues that aid in the annotation, diagnosis, and validation of the thermal data.

### 2.4 Dataset annotation

Each cat in the dataset is assigned a unique identifier linking its digital and thermal images. The following metadata were recorded for each entry:

• Image type (thermal or digital),
• Health state (healthy or sick),
• Environmental setting (controlled or uncontrolled),
• Time of capture (day or night for uncontrolled settings).

The health classification (healthy vs. sick) was confirmed by licensed veterinarians based on clinical assessments. The final dataset includes 2,426 images:

• 1,899 thermal images
• 527 digital images

A full description of the TICD is explain in Table 1A, while Table 1B shows the full details of the dataset and how every cat is assigned an ID and shows all its relevant statistics.

**Table 1:** The upper part **(A)** shows the specification and full description of the dataset, while the lower part **(B)** shows full details of the dataset and how every cat is assigned an ID and shows all its relevant statistics.

(A) Data specification table							
Item	Description						
Subject area or application area	Thermal Imaging, AI Diagnostics, Veterinary Health, Pet Health Monitoring						
Specific application area	Detection of sick cats, Health monitoring, Image processing, Dataset creation for AI training						
Type of data	Thermal images, Digital images						
Data format	JPG (Joint Photographic Experts Group)						
Image Resolution	Thermal: 640 × 480 pixels						
	Digital: 640 × 480 pixels						
How data were acquired	Data manually collected using Seek Shot thermal imaging camera						
Distance from Subjects	Images captured at optimal distances to ensure clarity and detail						
Experimental factors	Indoor (controlled environment) and outdoor (uncontrolled environment), variations in temperature and lighting conditions, separate times of day						
Noise and Background	Controlled environments to minimize noise; clear backgrounds in many images						
Health Status Annotation	Images labelled as “Healthy” or “Sick”						
Annotations and Metadata	Extensive metadata including cat ID, environment, time of capture, health status.						
Description of data collection sample	Images include both healthy and sick cats, categorized by ID and image type (thermal or digital)						
Experimental features	Total images: 2,426 (1,899 thermal, 527 digital)						
Number of Cats	94 unique cats (81 healthy, 13 sick)						
Data source location	Indoor: Black Forest Pets and Supplies, Jeddah, Pet Oasis Al Batarji, Cat Lounge, Jeddah, Al-Asr Veterinary Doctors Center for Primary Care, Medina, Nabd Al Farah Veterinary Clinic, Medina; Outdoor: Al Ruwais, Jeddah, Al-Usbah District, Medina						
Data accessibility	The dataset mentioned in this article is uploaded by authors on figshare repository and publicly available at: https://doi.org/10.6084/m9.figshare.28926584						
Thermal image details	Color-coded images where each color represents a specific temperature in Celsius						

(B) Statistics of the Dataset collection information and its classification as per Cat ID							
Time	S. No.	Cat ID	# Digital Images	# Thermal Images	Health Status	Environment	Location
N/A	1	1	13	14	Healthy	Controlled	Indoor
	2	2	7	13	Healthy	Controlled	Indoor
	3	3	9	16	Healthy	Controlled	Indoor
	4	4	5	13	Healthy	Controlled	Indoor
	5	5	7	12	Healthy	Controlled	Indoor
	6	6	9	2	Healthy	Controlled	Indoor
	7	7	4	11	Healthy	Controlled	Indoor
	8	8	9	7	Healthy	Controlled	Indoor
	9	9	7	4	Healthy	Controlled	Indoor
	10	10	8	7	Healthy	Controlled	Indoor
	11	11	5	12	Healthy	Controlled	Indoor
	12	12	7	20	Healthy	Controlled	Indoor
	13	13	4	8	Healthy	Controlled	Indoor
	14	14	6	10	Healthy	Controlled	Indoor
	15	15	9	10	Healthy	Controlled	Indoor
	16	16	11	10	Healthy	Controlled	Indoor
	17	17	10	10	Healthy	Controlled	Indoor
	18	18	9	9	Healthy	Controlled	Indoor
	19	19	10	10	Healthy	Controlled	Indoor
	20	20	10	10	Healthy	Controlled	Indoor
	21	21	10	10	Healthy	Controlled	Indoor
	22	22	10	10	Healthy	Controlled	Indoor
	23	23	10	10	Healthy	Controlled	Indoor
	24	24	10	11	Healthy	Controlled	Indoor
	25	25	10	9	Healthy	Controlled	Indoor
	26	26	3	2	Healthy	Controlled	Indoor
	27	27	4	4	Healthy	Controlled	Indoor
	28	28	1	1	Healthy	Controlled	Indoor
	29	29	4	4	Healthy	Controlled	Indoor
	30	30	1	1	Healthy	Controlled	Indoor
	31	31	2	2	Healthy	Controlled	Indoor
	32	32	1	1	Healthy	Controlled	Indoor
	33	33	1	1	Healthy	Controlled	Indoor
	34	34	1	1	Healthy	Controlled	Indoor
	35	35	1	1	Healthy	Controlled	Indoor
	36	36	1	1	Healthy	Controlled	Indoor
	37	37	1	1	Healthy	Controlled	Indoor
	38	38	1	1	Healthy	Controlled	Indoor
	39	39	1	1	Healthy	Controlled	Indoor
	40	40	2	2	Healthy	Controlled	Indoor
	41	41	1	1	Healthy	Controlled	Indoor
	42	42	4	4	Healthy	Controlled	Indoor
	43	43	1	1	Healthy	Controlled	Indoor
	44	44	1	1	Healthy	Controlled	Indoor
	45	45	9	12	Healthy	Controlled	Indoor
	46	46	11	9	Healthy	Controlled	Indoor
	47	48	10	4	Healthy	Controlled	Indoor
	48	61	1	15	Healthy	Controlled	Indoor
	49	62	5	15	Healthy	Controlled	Indoor
	50	63	6	26	Healthy	Controlled	Indoor
	51	64	7	41	Healthy	Controlled	Indoor
	52	65	4	7	Healthy	Controlled	Indoor
	53	66	8	41	Healthy	Controlled	Indoor
	54	67	8	58	Healthy	Controlled	Indoor
	55	68	4	57	Healthy	Controlled	Indoor
	56	69	2	11	Healthy	Controlled	Indoor
	57	70	2	44	Healthy	Controlled	Indoor
	58	71	4	32	Healthy	Controlled	Indoor
	59	73	6	58	Healthy	Controlled	Indoor
	60	74	4	51	Healthy	Controlled	Indoor
	61	75	2	19	Healthy	Controlled	Indoor
	62	76	9	50	Healthy	Controlled	Indoor
	63	77	4	88	Healthy	Controlled	Indoor
	64	78	2	17	Healthy	Controlled	Indoor
	65	47	10	10	Sick	Controlled	Indoor
	66	49	10	10	Sick	Controlled	Indoor
	67	90	12	94	Sick	Controlled	Indoor
	68	91	12	21	Sick	Controlled	Indoor
	69	92	11	19	Sick	Controlled	Indoor
	70	93	11	89	Sick	Controlled	Indoor
	71	94	10	84	Sick	Controlled	Indoor
	72	95	12	62	Sick	Controlled	Indoor
	73	96	14	94	Sick	Controlled	Indoor
	74	97	11	88	Sick	Controlled	Indoor
	75	98	15	93	Sick	Controlled	Indoor
Day	76	53	1	1	Healthy	Uncontrolled	Outdoor
	77	54	1	1	Healthy	Uncontrolled	Outdoor
	78	55	1	1	Healthy	Uncontrolled	Outdoor
	79	56	1	1	Healthy	Uncontrolled	Outdoor
	80	58	1	1	Healthy	Uncontrolled	Outdoor
	81	59	1	1	Healthy	Uncontrolled	Outdoor
	82	60	1	2	Healthy	Uncontrolled	Outdoor
	83	57	1	1	Sick	Uncontrolled	Outdoor
	84	89	10	88	Sick	Uncontrolled	Outdoor
Night	85	79	3	12	Healthy	Uncontrolled	Outdoor
	86	80	3	16	Healthy	Uncontrolled	Outdoor
	87	81	5	18	Healthy	Uncontrolled	Outdoor
	88	82	1	14	Healthy	Uncontrolled	Outdoor
	89	83	2	7	Healthy	Uncontrolled	Outdoor
	90	84	2	11	Healthy	Uncontrolled	Outdoor
	91	85	1	5	Healthy	Uncontrolled	Outdoor
	92	86	1	6	Healthy	Uncontrolled	Outdoor
	93	87	3	4	Healthy	Uncontrolled	Outdoor
	94	88	1	10	Healthy	Uncontrolled	Outdoor
	95	89	10	91	Sick	Uncontrolled	Outdoor

## 3 Potential of the TICD

The dataset supports downstream applications such as segmentation, temperature-based feature extraction, and classification of health states (healthy vs. sick) using machine learning and deep learning models (e.g., CNNs).

Figure 1B illustrates a workflow for classifying the health status of a cat (sick or healthy) using the TICD dataset. The process leverages image processing techniques combined with machine learning (ML) or deep learning (DL) classifiers. The workflow outlines the *training process* for a cat health classification model using thermal images from the TICD dataset. The workflow begins with a thermal image of a cat, which undergoes segmentation to isolate the animal from the background. A temperature histogram is generated from segmented image, providing key thermal statistics (average, min, max, most frequent temperature). These statistics are then compiled in a feature vector.

This features vector (or potentially the segmented image directly, if a CNN is used) is fed into an ML/DL classifier (e.g., a Deep Learning Classifier like a CNN). During the training phase, this classifier learns to associate specific temperature patterns or image features with the corresponding health state (sick or healthy) of the cat. The goal of this training is to build a robust model that can accurately predict a cat's health status from new thermal images.

## 4 TICD baseline

To establish a baseline for future research and provide a lower bound for comparison, we implemented the pipeline illustrated in Figure 1B. In this approach, the input thermal image undergoes a series of processing steps: segmentation, temperature histogram generation, feature vector extraction, and finally, classification using a machine learning model, logistic regression in our case. The model then outputs the predicted class labels.

The segmentation technique utilizes region masking and binary thresholding. First, specific areas such as the color bar, borders, and irrelevant regions are masked out using Boolean arrays. The remaining region is then converted to grayscale and thresholded to create a binary mask that isolates the cat's body. This segmented region is subsequently used to extract relevant thermal data, minimizing interference from background noise or overlay graphics.

After segmentation, the histogram generation module uses OCR to extract temperature scale values from the color bar, maps pixel colors to temperature values using linear interpolation, and calculates key statistical features. These include average temperature, minimum temperature, maximum temperature, and most frequent temperature, which serve as informative indicators of potential thermal anomalies related to cat's health status.

The dataset is divided into training and testing sets, with 80% allocated for training and 20% for testing. The logistic regression model achieved an accuracy of 73.42%, a specificity of 75.96%, a precision of 67.88%, a recall of 69.92%, and an F1 score of 68.89%.

## 5 Discussion

The TICD dataset includes only two labels: *Sick* and *Healthy*. The *Sick* label serves as a general indicator of illness, without specifying the cause, specific health condition, or symptoms. In its current form, TICD only indicates whether a cat is sick or not. The health labels in the TICD dataset are assigned based on joint confirmation by both the cat's owner and a licensed veterinarian. In all cases labeled as *sick*, the owner first reported signs of illness or abnormal behavior, which is clinically evaluated and confirmed by the veterinarian. This binary classification of *Sick* or *Healthy* represents a basic or initial level of investigation. In future iterations of the dataset, it can be extended to include more detailed information, such as the specific disease diagnosis and accompanying symptoms.

Although the TICD dataset includes 81 healthy cats and only 13 sick cats, resulting in a total of 1,899 thermal images, sick cats are captured more frequently per individual, contributing to 44.44% of the images, while healthy cats account for 55.56%. Despite this more balanced image-level distribution, the underlying class imbalance at the individual (cat) level can still impact machine learning performance. Specifically, models trained on such imbalanced data may become biased toward the majority class (healthy), leading to reduced sensitivity and a higher rate of false negatives when identifying sick cases. This issue especially concerns health-related applications, where accurately detecting abnormal or pathological conditions is critical. To help researchers address this imbalance, we recommend the following strategies:

• Class Rebalancing: Applying techniques such as under-sampling the majority class or over-sampling the minority class can help balance the dataset and improve model sensitivity to underrepresented classes.
• Synthetic Data Augmentation: Generating new samples for the minority class using methods like SMOTE (Synthetic Minority Over-sampling Technique) or employing image augmentation techniques (e.g., rotation, translation, flipping) can enhance data diversity and help models generalize better.
• Do not rely on accuracy alone. Use metrics that reflect performance on the minority class, like Precision, Recall, F1-score.
• Ensemble Methods: Using ensemble models that are more robust to imbalance classes like: Balanced Random Forest, XGBoost and LightGBM allow custom class weights.
• By applying these strategies, researchers can develop more balanced and reliable classification models, making full use of the TICD dataset's potential for non-invasive health monitoring of cats.

## Data Availability

The datasets presented in this study can be found in online repositories. The names of the repository/repositories and accession number(s) can be found below: https://doi.org/10.6084/m9.figshare.28926584.
