# Supplementary material for: TICD: a novel thermal imaging cats' dataset for non-invasive health monitoring
Source: Front Digit Health. 2025 Aug 15;7:1650223. doi: 10.3389/fdgth.2025.1650223 (PMC12394521; doi:10.3389/fdgth.2025.1650223)
Supplement: Supplementary file 1 [file Datasheet1.pdf]

Code for Main module of Baseline Implementation

Segmentation Module:

```
from PIL import Image

import matplotlib.pyplot as plt

import numpy as np

import os

import cv2 as cv

# Define input and output directories for cat thermal images

ifolder = r'Thermal Image Dataset of cats for their health monitoring
and classification\Controlled_environment_(Indoor)\Sick\Thermal\Cat_ID_97'

ofolder = r'segmented\sick\Cat_ID_97'


os.makedirs(ofolder, exist_ok=True)

# List all JPEG files in the input folder

file_list = [f for f in os.listdir(ifolder) if f.endswith('.jpg')]

# Define coordinates for masking the color bar and other non-relevant regions

color_bar_x = [550, 640]

color_bar_y = [30, 450]

top_part_x = [0, 640]

top_part_y = [0, 50]

bottom_part_x = [0, 640]

bottom_part_y = [420, 480]

left_part_x = [0, 50]

left_part_y = [0, 480]

# Loop through each image for segmentation

for file_name in file_list:

  img_path = os.path.join(ifolder, file_name)

  img = Image.open(img_path)

  img_array = np.array(img)
```

```
    # Convert image to grayscale and apply region masking
    img_gray = cv.cvtColor(img_array, cv.COLOR_RGB2GRAY)
    color_bar_mask = np.ones_like(img_gray, dtype=bool)
    color_bar_mask[color_bar_y[0]:color_bar_y[1], color_bar_x[0]:color_bar_x[1]] = False
    top_part_mask = np.ones_like(img_gray, dtype=bool)
    top_part_mask[top_part_y[0]:top_part_y[1], top_part_x[0]:top_part_x[1]] = False
    bottom_part_mask = np.ones_like(img_gray, dtype=bool)
    bottom_part_mask[bottom_part_y[0]:bottom_part_y[1], bottom_part_x[0]:bottom_part_x[1]] = 
False
    left_part_mask = np.ones_like(img_gray, dtype=bool)
    left_part_mask[left_part_y[0]:left_part_y[1], left_part_x[0]:left_part_x[1]] = False
    # Combine masks and apply thresholding to obtain the binary mask
    img_gray_masked = img_gray * color_bar_mask * bottom_part_mask * top_part_mask * 
left_part_mask
    thres = 29  # Adjust threshold as needed
    maxVal = 255
    _, imgThres = cv.threshold(img_gray_masked, thres, maxVal, cv.THRESH_BINARY)
    binary_mask = imgThres > 0
    # Apply the binary mask to the original image to segment the ROI
    thermal_cat_only = np.zeros_like(img_array)
    for channel in range(img_array.shape[2]):
        thermal_cat_only[..., channel] = img_array[..., channel] * binary_mask
    # Save and display the segmented image
    output_path = os.path.join(ofolder, f"{file_name}_segmented.jpg")
    Image.fromarray(thermal_cat_only.astype(np.uint8)).save(output_path)
    plt.figure(figsize=(10, 5))
    plt.title("Thermal Image with Masked Color Bar and Applied Binary Mask")
    plt.imshow(thermal_cat_only)
    plt.axis('off')
    plt.tight_layout()
    plt.show()
```

Histogram Generation Module:

```
import os

from PIL import Image

import numpy as np

import matplotlib.pyplot as plt

import pytesseract

import cv2

import csv


# Define folder paths for thermal images, segmented images and output histograms

thermal_images_folder = r'Thermal Image Dataset of cats for their health monitoring and classification\Controlled_environment_(Indoor)\Sick\Thermal\Cat_ID_96'

segmented_images_folder = r'segmented\sick\Cat_ID_96'

output_folder = r'histogram\sick\Cat_ID_96'


# Ensure output folder exists

os.makedirs(output_folder, exist_ok=True)


# List all thermal and segmented images

thermal_image_files = os.listdir(thermal_images_folder)

segmented_image_files = os.listdir(segmented_images_folder)


# Color bar position

color_bar_x = [605, 620]

color_bar_y = [72, 410]
```

```
# Temperature text positions
lowest_temp_pos = {'top_left': (580, 420), 'bottom_right': (630, 450)}
highest_temp_pos = {'top_left': (580, 30), 'bottom_right': (630, 60)}


def extract_color_bar(image, x_range, y_range):
    """Extract the color bar from the image."""
    return image[y_range[0]:y_range[1], x_range[0]:x_range[1]]


def extract_temperature(image, pos):
    """Extract temperature value from the specified position using OCR."""
    x1, y1 = pos['top_left']
    x2, y2 = pos['bottom_right']
    roi = image[y1:y2, x1:x2]
    roi = cv2.cvtColor(roi, cv2.COLOR_BGR2GRAY)
    roi = cv2.threshold(roi, 0, 255, cv2.THRESH_BINARY | cv2.THRESH_OTSU)[1]
    custom_config = r'--oem 3 --psm 6 outputbase digits'
    temperature = pytesseract.image_to_string(roi, config=custom_config)
    return float(temperature.strip())


def display_color_bar_with_temperatures(color_bar, min_temp, max_temp):
    """Display the color bar with temperature labels."""
    bar_height = color_bar.shape[0]
    plt.figure(figsize=(2, 6))
    plt.imshow(color_bar, aspect='auto', extent=[0, 1, min_temp, max_temp])
```

```
    plt.title("Color Bar with Temperature Mapping")
    plt.xlabel("Color")
    plt.ylabel("Temperature (°C)")
    plt.yticks(np.linspace(min_temp, max_temp, num=5))  # Add temperature ticks
    plt.show()

def create_color_to_temp_mapping(color_bar, min_temp, max_temp):
    """Create a mapping from color to temperature based on the color bar."""
    color_to_temp = {}
    bar_height = color_bar.shape[0]
    temp_range = max_temp - min_temp
    for i in range(bar_height):
        color = tuple(color_bar[i, 0])  # Assuming the color bar is vertical and 1 pixel wide
        temperature = min_temp + (i / bar_height) * temp_range
        color_to_temp[color] = temperature
    return color_to_temp

def apply_color_mapping(image, color_to_temp):
    """Apply the color to temperature mapping to the image."""
    temp_image = np.zeros(image.shape[:2])
    for i in range(image.shape[0]):
        for j in range(image.shape[1]):
            color = tuple(image[i, j])
            if color in color_to_temp:
                temp_image[i, j] = color_to_temp[color]
```

```
    return temp_image


def display_temperature_histogram(temp_image, min_temp, max_temp, average_temp=None, min_temp_roi=None, max_temp_roi=None, most_frequent_temp=None):

    """Display a histogram of the number of pixels at each temperature."""

    plt.figure(figsize=(12, 6))

    plt.subplot(131)

    plt.hist(temp_image[temp_image > 0].flatten(), bins=50, range=(min_temp, max_temp), color='blue', alpha=0.7)



    # Add vertical lines for min, max, average, and most frequent temperatures

    if average_temp is not None:

        plt.axvline(average_temp, color='red', linestyle='dashed', linewidth=2, label=f'Average Temp: {average_temp:.2f} °C')

    if min_temp_roi is not None:

        plt.axvline(min_temp_roi, color='green', linestyle='dashed', linewidth=2, label=f'Min Temp: {min_temp_roi:.2f} °C')

    if max_temp_roi is not None:

        plt.axvline(max_temp_roi, color='purple', linestyle='dashed', linewidth=2, label=f'Max Temp: {max_temp_roi:.2f} °C')

    if most_frequent_temp is not None:

        plt.axvline(most_frequent_temp, color='orange', linestyle='dashed', linewidth=2, label=f'Most Frequent Temp: {most_frequent_temp:.2f} °C')



    plt.legend()

    plt.title("Temperature Histogram")

    plt.xlabel("Temperature (°C)")

    plt.ylabel("Number of Pixels")
```

```
    plt.grid(True)

    plt.subplot(132)
    plt.imshow(thermal_array)
    plt.title(f"Thermal Image: {thermal_image_file}")

    plt.subplot(133)
    plt.imshow(segmented_array)
    plt.title(f"Segmented Image")

    # Save the temperature-mapped image
    output_file = os.path.join(output_folder, thermal_image_file + '_histogram.jpg')
    plt.savefig(output_file)

    plt.show()

def calculate_most_frequent_temperature(temp_image):
    """Calculate the most frequent temperature in the temperature-mapped image."""
    # Exclude background pixels (assuming background is 0)
    roi_pixels = temp_image[temp_image > 0]
    if roi_pixels.size == 0:
        print("No valid pixels found in the segmented region.")
        return None

    # Find the most frequent temperature
```

```
    unique_temps, counts = np.unique(roi_pixels, return_counts=True)
    most_frequent_temp = unique_temps[np.argmax(counts)]
    return most_frequent_temp

def calculate_average_temperature(temp_image):
    """Calculate the average temperature of the segmented region."""
    # Exclude background pixels (assuming background is 0)
    roi_pixels = temp_image[temp_image > 0]
    if roi_pixels.size == 0:
        print("No valid pixels found in the segmented region.")
        return None
    average_temp = np.mean(roi_pixels)
    return average_temp

def calculate_min_max_temperature(temp_image):
    """Calculate the minimum and maximum temperatures of the segmented region."""
    # Exclude background pixels (assuming background is 0)
    roi_pixels = temp_image[temp_image > 0]
    if roi_pixels.size == 0:
        print("No valid pixels found in the segmented region.")
        return None, None
    min_temp = np.min(roi_pixels)
    max_temp = np.max(roi_pixels)
    return min_temp, max_temp
```

```
def save_histogram_info_to_csv(output_csv_file, histogram_info):
    """
    Save histogram information to a CSV file.

    Parameters:
        output_csv_file (str): Path to the output CSV file.
        histogram_info (list): List of dictionaries containing histogram information for each image.
    """
    # Define the CSV column headers
    fieldnames = ["Image Name", "Average Temperature", "Min Temperature", "Max Temperature", "Most Frequent Temperature"]

    # Write the histogram information to the CSV file
    with open(output_csv_file, mode='w', newline='') as csv_file:
        writer = csv.DictWriter(csv_file, fieldnames=fieldnames)

        # Write the header row
        writer.writeheader()

        # Write the data rows
        for info in histogram_info:
            writer.writerow(info)

    print(f"Histogram information saved to {output_csv_file}")
```

```
# Main loop
histogram_info = []  # List to store histogram information for all images


for thermal_image_file in thermal_image_files:
    # Get the first 5 characters of thermal image filename
    thermal_prefix = thermal_image_file[:5]


    # Find matching segmented image
    matching_segmented_files = [f for f in segmented_image_files if 
f.startswith(thermal_prefix)]
    if not matching_segmented_files:
        print(f"No matching segmented image found for {thermal_image_file}. Skipping.")
        continue
    else:
        # Assuming there's only one matching file
        segmented_image_file = matching_segmented_files[0]


    thermal_image_path = os.path.join(thermal_images_folder, thermal_image_file)
    segmented_image_path = os.path.join(segmented_images_folder, segmented_image_file)


    # Open and show thermal image to the user
    thermal_image = Image.open(thermal_image_path)
    thermal_array = np.array(thermal_image)


    # Extract lowest and highest temperatures using OCR
    min_temp = extract_temperature(thermal_array, lowest_temp_pos)
```

```
max_temp = extract_temperature(thermal_array, highest_temp_pos)

# Display the thermal image
plt.imshow(thermal_array)
plt.title(f"Thermal Image: {thermal_image_file}")
plt.axis('off')
plt.show()

# Extract color bar from thermal image
color_bar = extract_color_bar(thermal_array, color_bar_x, color_bar_y)

# Display the color bar with temperature mapping
display_color_bar_with_temperatures(color_bar, min_temp, max_temp)

# Open segmented image and convert to grayscale
segmented_image = Image.open(segmented_image_path).convert('RGB')
segmented_array = np.array(segmented_image)

# Create color to temperature mapping
color_to_temp = create_color_to_temp_mapping(color_bar, min_temp, max_temp)

# Apply color mapping to segmented image
temp_image = apply_color_mapping(segmented_array, color_to_temp)

# Calculate the average temperature
```

```
    average_temp = calculate_average_temperature(temp_image)

    # Calculate the min and max temperatures
    min_temp_roi, max_temp_roi = calculate_min_max_temperature(temp_image)

    # Calculate the most frequent temperature
    most_frequent_temp = calculate_most_frequent_temperature(temp_image)

    # Display the results
    if average_temp is not None and min_temp_roi is not None and max_temp_roi is not None and most_frequent_temp is not None:
        print(f"Temperature Statistics for {thermal_image_file}:")
        print(f"  Minimum Temperature: {min_temp_roi:.2f} °C")
        print(f"  Maximum Temperature: {max_temp_roi:.2f} °C")
        print(f"  Average Temperature: {average_temp:.2f} °C")
        print(f"  Most Frequent Temperature: {most_frequent_temp:.2f} °C")
    else:
        print(f"Could not calculate temperature statistics for {thermal_image_file}.")

    # Store histogram information for this image
    histogram_info.append({
        "Image Name": thermal_image_file,
        "Average Temperature": average_temp,
        "Min Temperature": min_temp_roi,
        "Max Temperature": max_temp_roi,
        "Most Frequent Temperature": most_frequent_temp
```

```
    })

    # Display the temperature histogram with min, max, average, and most frequent temperatures

    display_temperature_histogram(temp_image, min_temp, max_temp, average_temp, min_temp_roi, max_temp_roi, most_frequent_temp)

    # Save histogram information to a CSV file

output_csv_file = os.path.join(output_folder, "histogram_info.csv")

save_histogram_info_to_csv(output_csv_file, histogram_info)
```

Logistic Regression Model Code:

```
import pandas as pd

from sklearn.preprocessing import LabelEncoder

from sklearn.model_selection import train_test_split

from sklearn.metrics import accuracy_score, confusion_matrix, ConfusionMatrixDisplay

import matplotlib.pyplot as plt

from sklearn.linear_model import LogisticRegression


# Load and clean dataset

data = pd.read_csv(r"histogram\alldata.csv")

data['class'] = data['class'].str.strip()

data = data[data['class'].isin(['Sick', 'Healthy'])]


print("Dataset Information:")

print(f"Total samples: {len(data)}")

print("Class distribution:")

print(data['class'].value_counts())

print()


# Encode labels

label_encoder = LabelEncoder()
```

```
data['class'] = label_encoder.fit_transform(data['class'])

# Select features and split data
X = data[['Average Temperature', 'Min Temperature', 'Max Temperature', 'Most Frequent Temperature']]
y = data['class']
X_train, X_test, y_train, y_test = train_test_split(X, y, test_size=0.2, random_state=42, stratify=y)

# Print sample counts after split
print("Data Split Information:")
print(f"Training samples: {len(X_train)} ({len(X_train)/len(data)*100:.1f}%)")
print(f"Testing samples: {len(X_test)} ({len(X_test)/len(data)*100:.1f}%)\n")

# Class distribution
print("Training set class distribution:")
for label, count in pd.Series(y_train).value_counts().items():
  print(f"  {label_encoder.inverse_transform([label])[0]}: {count}")

print("\nTesting set class distribution:")
for label, count in pd.Series(y_test).value_counts().items():
  print(f"  {label_encoder.inverse_transform([label])[0]}: {count}")
print()

# Define and train model(s)
models = {
  "Logistic Regression": LogisticRegression(random_state=42, solver='liblinear'),
}

for name, model in models.items():
  model.fit(X_train, y_train)
  y_pred = model.predict(X_test)
```

```
accuracy = accuracy_score(y_test, y_pred)
print(f"{name} - Accuracy: {accuracy:.4f}")

cm = confusion_matrix(y_test, y_pred)

if cm.shape == (2, 2):
    TN, FP, FN, TP = cm.ravel()
    print(f"\nModel: {name} (Accuracy: {accuracy:.4f})")
    print("Detailed Model Metrics:")
    print(f"True Positives (TP): {TP}")
    print(f"True Negatives (TN): {TN}")
    print(f"False Positives (FP): {FP}")
    print(f"False Negatives (FN): {FN}")

    precision = TP / (TP + FP) if (TP + FP) > 0 else 0
    recall = TP / (TP + FN) if (TP + FN) > 0 else 0
    specificity = TN / (TN + FP) if (TN + FP) > 0 else 0
    f1 = 2 * (precision * recall) / (precision + recall) if (precision + recall) > 0 else 0

    print(f"Precision: {precision:.4f}")
    print(f"Recall (Sensitivity): {recall:.4f}")
    print(f"Specificity: {specificity:.4f}")
    print(f"F1-Score: {f1:.4f}")
else:
    print(f"Model: {name}")
    print("Confusion Matrix shape:", cm.shape)
    print("Confusion Matrix:")
    print(cm)

# Plot confusion matrix
plt.figure(figsize=(8, 6))
```

```
    disp = ConfusionMatrixDisplay(confusion_matrix=cm,
display_labels=label_encoder.classes_)
    disp.plot(cmap=plt.cm.Blues, values_format='d')
    plt.title(f"Confusion Matrix - {name}\nAccuracy: {accuracy:.4f}")
    plt.tight_layout()
    plt.show()
```
